# Perceived Trauma Among Nurses During the COVID‐19 Pandemic: A Qualitative Descriptive Study

**DOI:** 10.1111/inm.70031

**Published:** 2025-03-26

**Authors:** Dianna Burr, Louise Alexander, Adam Searby

**Affiliations:** ^1^ School of Nursing & Midwifery Monash University Melbourne Australia; ^2^ Institute for Health Transformation, School of Nursing & Midwifery Deakin University Geelong Australia

**Keywords:** COVID‐19 pandemic, health workforce, nurses, nursing staff, professional burnout, psychological burnout, psychological trauma

## Abstract

The COVID‐19 pandemic has caused disruption to healthcare services worldwide, and although the pandemic was declared over, nurses continue to experience burnout and mental health implications because of events experienced since 2020. The aim of this study was to explore the experiences of trauma among nurses during the COVID‐19 pandemic, using a qualitative descriptive study design. This paper used secondary analysis of qualitative, semi‐structured interviews conducted with 55 Australian nurses exploring their alcohol consumption, due to the frequent narratives of trauma and traumatic events arising during this process. Data were analysed using structural coding and reported in accordance with the Consolidated Checklist for Reporting Qualitative Data (CORE‐Q guidelines). Four themes emerged during data analysis: managing the traumatic stress of the clinical environment in COVID (‘it doesn't feel like it's gone away’), post‐pandemic trauma (‘it was like war, wasn't it?’), bonding over shared trauma (‘I was not expecting the camaraderie that developed’) and managing trauma after the event (‘If something bad happens whilst you're on shift, you just have to pretend like it hasn't happened’). Participants in this study described lasting mental health effects from working clinically during the COVID‐19 pandemic that they described as trauma. These effects have lasted since governments worldwide have announced an end to the pandemic, and although participants described bonding over these shared experiences, they continue to have a detrimental impact on workforce well‐being and sustainability.

## 1 Introduction

The COVID‐19 pandemic touched all corners of the globe and has continued to do so since it was first identified in 2019 (World Health Organisation 2024). To date, COVID‐19 has claimed over seven million lives (World Health Organisation 2024) including an estimated 180 000 deaths of healthcare workers (World Health Organisation 2021). Nurses were particularly vulnerable to both infection from the COVID‐19 virus and the psychological impacts of being a frontline worker during an unprecedented pandemic (Shaukat et al. 2020). The loss of healthcare workers not only creates additional burdens on an already stretched system but also impacts the psychological well‐being of healthcare staff, who are constantly reminded of the personal risk they take in simply doing their job (Shreffler et al. 2020).

The global impacts of COVID‐19 on healthcare workers were reported in studies identifying higher rates of acute stress disorder (Xiao et al. 2020), vicarious trauma (Li et al. 2020) and traumatic stress (Chew et al. 2020; Kang et al. 2020; Lai et al. 2020; Tan et al. 2020; Zhang et al. 2020). While COVID‐19's impacts were consistently difficult for healthcare workers worldwide, one region was additionally affected by significant lockdowns; Australia was in lockdown for 6 weeks at the start of the pandemic, and this was followed by significant restrictions in the state of Victoria, where 16 successive weeks of lockdown occurred (Australian Bureau of Statistics 2021). While the mental health of nurses was widely reported as poor with higher rates of anxiety, stress, depression, post‐traumatic stress disorder and insomnia (Varghese et al. 2021), little is understood about the traumatic experiences of nurses in Australia during the pandemic. Therefore, the aim of this study was to explore perceived trauma among nurses both during the COVID‐19 pandemic and after the declared end of the pandemic.

## 2 Background

When the first Australian case of COVID‐19 was announced in the state of Victoria on 25 January 2020 (Parliament of Australia 2020), few foresaw what would ensue over the next few years. Healthcare workers were exposed to the risk of infection, significant changes to workplace function and policy, stress and increasing hostility from the public. Already familiar with occupational violence and aggression (OVA), healthcare workers endured increased OVA during the pandemic, which continued to be a stressor with significant detrimental impacts to healthcare workers mental health (Tiesman et al. 2023). While the rates of workplace violence experienced by American healthcare workers increased during the pandemic (Ward et al. 2022), Topazian et al. (2022) also identified that 21% of a representative survey of 1086 American adults believed hostility towards healthcare workers was justified because of business closures.

Over the course of the past 4 years of the pandemic, there was significant international research on the physical and mental health and well‐being of healthcare workers, with reports of high rates of stress, fear, anxiety, depression and insomnia (Biber et al. 2022; Shreffler et al. 2020; Smallwood et al. 2022). As frontline workers, nurses were required to work with ongoing uncertainty, whilst also managing high acuity and workloads, often without sufficient equipment to do this safely (Ayton et al. 2022; Qureshi et al. 2022). Stress in nurses was exacerbated by policy changes that were inadequately funded, coupled with significant resource shortages and healthcare systems that were undermined, resulting in higher rates of burnout and adverse mental health outcomes (Boyden and Brisbois 2023).

Traumatic stress in nurses during the pandemic was consistently reported in the literature (Benfante et al. 2020; d'Ettorre et al. 2021; Hernandez et al. 2021), however, trauma in nurses is not well understood. Understanding and responding to trauma in nurses is important because, like anyone who is traumatised, they may be more prone to engage in maladaptive coping strategies to manage their experiences; Foli et al. (2021) found that nurses were considerably traumatised and consequently increased their use of substances to cope. Foli et al. (2021) also identified that a negative impact on their traditional coping supports resulted in maladaptive coping strategies such as alcohol misuse. This is consistent with Searby et al. (2024), who identified that workplace pandemic stress resulted in an increase in alcohol consumption in Australian nurses. As such, it is important to develop an understanding of the impacts and coping mechanisms that nurses use in the face of trauma to develop strategies and supports for them.

Despite this upheaval, nurses did make efforts to moderate the distress they were experiencing. To mitigate the stress of working during the pandemic, international research revealed several different mechanisms nurses adopted, including venting and using humour (Sierakowska and Doroszkiewicz 2022). Kim et al. (2021) also found that resilience, spirituality and positive family functioning were identified by a sample of nurses from the United States as constructive coping mechanisms during times of pandemic stress. Despite the personal attributes that nurses can use to mitigate the stress they experience, cumulative stressors have a toll on the well‐being of nurses. Posttraumatic growth, defined as a process of adaptation in the presence of adversity where the resulting change is considered positive (Tedeschi and Calhoun 2004). Kalaitzaki et al. (2024) described an increase in posttraumatic growth measures in nurses who had been experiencing the pandemic impacts for several years. This process is supported by other research, which has described posttraumatic growth as a positive consequence of the pandemic for healthcare workers (Bai and Bai 2024; Feingold et al. 2022; Yeung et al. 2022).

## 3 Study Aim

The aim of this study was to explore perceived trauma among nurses both during the COVID‐19 pandemic and after the declared end of the pandemic.

## 4 Methods

### 4.1 Design

Qualitative description was used as the overarching design for this study. By definition, qualitative description is a naturalistic, low‐inference qualitative design where data are analysed and presented in the words of the participants (Neergaard et al. 2009). We used this design in conjunction with Structural Coding, a method of analysing data in accordance with the aims or research questions of the study (Saldaña 2013), to allow participant narratives of their own experiences of trauma to be presented with minimal interpretation by the authors.

### 4.2 Data Collection

Sampling for this study was conducted while participants were completing a broader survey of alcohol consumption among Australian nurses. Upon conclusion of the survey, participants were asked whether they wished to participate in a telephone interview exploring workplace stress and alcohol consumption. Prior to signing up, participants could view a Participant Information Sheet for the survey, and if agreeable, leave a contact email address for follow up by a research assistant experienced in qualitative data collection (Author 1). Of the 649 participants in the survey, 115 provided contact details for follow up by the research assistant. After initial contact, 55 responded to schedule an interview (approximately 48%). During these interviews, several accounts of trauma relating to the COVID‐19 pandemic were described, leading to a secondary analysis of the dataset in accordance with the research aim.

Interviews were conducted by the first author (D.B.) between October 2023 and January 2024. The mean duration of the interviews was 38:01, with a standard deviation of 8:51 and a range of 25:11–1:02:30. All participants who commenced an interview completed the interview, with no requests to cease the interview or withdraw data received. On completion of the interview, participants were reimbursed with a $50 eGift voucher redeemable at an approved online or physical retailer of their choice. We continued interviewing beyond theoretical data saturation to ensure a wide range of perspectives from nurses around Australia, and a wide variety of healthcare settings were recorded. All interviews were recorded verbatim and transcribed using the MacWhisper app, which uses the OpenAI Whisper Large Language Model to transcribe audio (Good Snooze, 2024). This app was used as it runs on a local computer rather than transferring data to cloud‐based servers, ensuring that sensitive participant information was never exposed to the internet, nor left Australia. All transcripts were checked by the second and third author (L.A. & A.S.) to ensure the transcript was an accurate rendition of the recorded audio.

The semi‐structured interview guide was based on the previous version of the project exploring nurses' alcohol consumption, conducted in Searby et al. (2021). This interview guide was used with 42 Australian nurses, and the guide was modified based on review of previous interview recordings, feedback from prior participants, and a scan of emerging literature on the nursing workforce 3 years on from the COVID‐19 pandemic.

### 4.3 Analysis

Secondary analysis of the dataset was performed by two members of the research team (Author 1 and Author 3). After transcriptions were prepared by the MacWhisper app, they were uploaded to NVivo version 14 for analysis (Lumivero, 2022). Saldaña's (2013) method of structural coding was used, a deductive approach where data is coded according to the research question guiding the study. In this instance, we analysed transcripts for participant descriptions of experiences and perceptions of workplace trauma occurring during the COVID‐19 pandemic; this process aligned with the first and second steps of the Braun and Clarke (2006) method and allowed generation of an initial coding framework recognising traumatic events. Remaining steps were conducted according to this method: (3) reviewing themes, (4) defining themes, (5) naming themes, and finally, (6) producing the report.

### 4.4 Findings

A total of 55 nurses took part in this study, 50 female and 5 males. Most participants were working in clinical nursing roles (*n* = 20, 36%) and held a mean number of years of clinical experience of 23.1 (standard deviation 11.8 years). Full participant demographic information, including work setting, is presented in Table 1. Four themes emerged during data analysis: managing the traumatic stress of the clinical environment in COVID (‘it doesn't feel like it's gone away’), post‐pandemic trauma (‘it was like war, wasn't it?’), bonding over shared trauma (‘I was not expecting the camaraderie that developed’) and managing trauma after the event (‘If something bad happens whilst you're on shift, you just have to pretend like it hasn't happened’).

**TABLE 1:** Participant demographics.

Characteristics	*n*	%^a^	*M*	SD
Gender				
Female	50	91%		
Male	5	9%		
Experience (years)			23.1	11.8
Role				
Clinical nurse	20	36%		
Nurse educator	19	35%		
Nurse practitioner	11	20%		
Nurse manager	4	7%		
Clinical nurse consultant	1	2%		
Work setting				
General hospital	9	16%		
Perioperative	8	15%		
Alcohol and other drugs	7	13%		
Emergency department	6	11%		
Mental health	6	11%		
Community	5	9%		
Maternity	2	4%		
Research	2	4%		
Disability	1	2%		
Intensive care unit	1	2%		
Paediatric	1	2%		
Critical care	1	2%		
Primary health	1	2%		
Cardiac	1	2%		
Outpatients	1	2%		
Palliative care	1	2%		
Policy	1	2%		
Sexual health	1	2%		

^a^ Totals may not equal 100% due to rounding.

#### 4.4.1 Managing the Traumatic Stress of the Clinical Environment: ‘it Doesn't Feel Like It's Gone Away’

The first key theme identified in this study explores nurses' experiences and attempts at managing the traumatic stress of the clinical environment, particularly during the COVID‐19 pandemic, and in the following years as the pandemic subsided. Largely, participants spoke of the clinical environment being unpredictable during the pandemic; however, since the pandemic was declared over in Australia, participants noted that the mental health impacts of the constantly changing clinical environment persisted, leading to a lasting traumatic feeling of ‘impending doom’:

… it doesn't quite feel like it's gone away. It's almost sometimes it's like there's a feeling of impending doom, but you're not sure what that doom is likely to look like. (Participant 1)

It is well documented that during the early days of the pandemic when little was known or understood about the virus, such as the method of transmission, healthcare organisations were largely in the dark regarding the most effective infection control management practices to be implemented to protect patients, healthcare workers and families. For nurses working in the clinical environment during this time, they describe how the daily constantly changing updates to infection control practices were a key contributing factor to their levels of traumatic stress:

… during the pandemic … you're in the N95 mask, which is just awful, for a whole shift. There's extra, you know, all the other PPE you've got to wear. The constant changing rules about what you do when there's a COVID patient or if they've been a close contact. (Participant 55)

For those nurses in more senior roles, their traumatic stress was related to the expectations they felt from junior staff that they would know what to do, what was coming and how to manage it. They described how staff would look to them for support and reassurance, and how stressful it was managing staff who were fearful as well as anxious about what to do for their patients who had COVID‐19 and how to do it safely:

Every day there were new rules from infection control, and you were expected to be on top of it all the time as well as dealing the nursing stuff, [the] unknown and their fear. The questions that they all had and seeing a senior nurse, you were expected to know everything. And almost any weakness that you show can come out quickly in your team so you're just trying to do your best. (Participant 43)

In that constantly changing environment many nurses described the fear that they had; fear of contracting COVID‐19 which was continually described as a significant source of stress, anxiety and worry while working clinically amidst the pandemic:

I just think it was a situation that was new to the world, and they were dealing with it the best way they could, but it was just this constant change and constant fear of, am I going to get COVID? (Participant 26)

For some the fear was exacerbated by not having access to the correct PPE, and just having to ‘make do with whatever was there’, (Participant 26) and for others fortunate enough to have access to that PPE it was simply coming face to face with this unknown new virus that was rapidly spreading throughout the country, causing severe illness and death, leading to fear and potential lasting trauma from working with this fear constantly present:

It was definitely scary … in the early days, it was fear of COVID. Because the whole country was in lockdown, but I was looking after people who had COVID in the eye every day, despite an awful lot of PPE. (Participant 31)

Participants also described experiencing the stress of professional trauma during the COVID‐19 pandemic when their core values of caring were challenged, with the unfolding situation preventing them being able to provide the nursing care they were accustomed to. Not being to provide the standard of care that nurses hold as defining their practice and even more importantly their identity was identified as being challenging, particularly where patients were dying from COVID‐19 and associated complications within the healthcare facilities they were working in:

Really stressful … I had a priest delivering last rites through a closed window from a garden bed outside the building … from a perspective of family, spiritual beliefs, people did not die well in that first two years, which made some of us feel like we weren't able to do our jobs properly. (Participant 11)

#### 4.4.2 Post Pandemic Trauma: ‘It Was Like War, Wasn't It?’

Participants in this study described the stress of experiences after the COVID‐19 pandemic and associated restrictions as a kind of trauma, with some even going so far as to describe it as Post Traumatic Stress Disorder (PTSD). Nursing tasks once considered benign are now forever associated with a time of great social upheaval and physical anxiety; activities associated with subsequent infectious outbreaks such as locking down a ward and a return to donning full PPE, now synonymous with the pandemic, became triggers that took them back to those times when they experienced intense feelings of stress and anxiety:

A couple of months ago, we had to have a full lockdown again. And it just kind of brought back all those memories of being in full PPE and the kind of a little bit of PTSD, I guess, during that time. (Participant 43)

Many months after the height of the pandemic, when the repeated lockdowns and travel restrictions had been lifted, participants reported experiencing ‘flashbacks’ when returning to normal activities that were reminiscent of previously distressing experiences. The following participant recounts reliving the anxieties and trauma of having to pass through the mandatory military type road blockade just to get home:

I drove down a road where I had sort of like a flashback to that was where there was like one of those Checkpoint Charlies where I was pulled over and had my license checked and there was army and police and, you know, I remember being scared to be pulled over to show my license as to where I was going. And I thought, for fuck's sake, I'm going to work … I'm a normal person. I'm doing nothing wrong, but I had a bit of paper saying that I live here and I'm going here. I remember being so scared that I had to go through this Checkpoint Charlie thing, you know, and it was like war, wasn't it? (Participant 9)

Many nurses spoke about fear as a driver for trauma, and for the following participant it was a realisation that the staff had not been able to satisfactorily deal with or manage the stresses and anxieties and traumas they had experienced during COVID‐19:

I think … when we had another lockdown episode … there was a lot of fear. People were reliving what it was like when we were a full lockdown ward. And that made me realise that there's trauma there. (Participant 43)

As well as reporting witnessed trauma directly relating to being ‘on the frontline’, participants described how the added pressures and demands of increases in staffing triggered undeclared instances of underlying trauma that had not been acknowledged, and the associated consequence of not being given the time or the opportunity to effectively deal with those experiences:

… these young nurses were so traumatised but then they got in trouble … they didn't want to do double night shifts and they had this big argument, and the nurses were angry that they weren't consulted. And it didn't take long to realise what they were really angry about was how they'd been treated and there was no acknowledgement of the trauma that they had witnessed. (Participant 22)

#### 4.4.3 Bonding Over Shared Experiences of Trauma: ‘I Was Not Expecting the Camaraderie That Developed’

Traditionally, nurses and other healthcare workers are given the opportunity to formally discuss unusual or distressing events or issues that occur in the course of their work by debriefing with senior staff or counsellors, whether at the time or at some time close to the event. It is also well understood that nurses will debrief informally together, whether one on one or as a group or team depending on the circumstances—conversations that are more likely to be had with colleagues rather than family members, as described by the following participant:

The way that we still managed to communicate with each other and still express our concern for each other and the teamwork between those two teams, it blew me away. I was not expecting the camaraderie that developed. It was absolutely fantastic. (Participant 30)

This camaraderie that bonds nurses with colleagues following a distressing event is fundamental to the way they process and deal with situations that enable them to ‘get back on the horse’, because as the following participant identifies, only nurses really understand what other nurses experience:

If we have a baby that dies or a mother that dies in childbirth or something traumatic, then they will offer [counselling] as a response to that. But not in a week‐by‐week sort of way. And so, I think nurses have always had very much an oral culture and so nurses talk, they just talk and support each other that way. (Participant 33)

However, during the COVID‐19 pandemic when the need to debrief face to face was arguably greater than usual, participants reported feeling increased levels of stress when isolated by furlough practices and imposed social isolating. Debriefing and networking was reported to be commonly done via technologies such as Zoom and Teams, as well as being used to facilitate interactions with patients (Telehealth). Whilst providing a platform for connection, several participants reported that it did not meet the same needs as the face‐to‐face experience:

But not being able to come back into the office at the end of the day and just go, I've had a really crappy day and or I had this problem, and I didn't know what to do and do you have any advice … all of that made a huge difference because … I think for nurses, that's how we manage the stresses of our work is to debrief with each other. And it's not the same when you're doing it over a video screen or email. (Participant 29)

For these participants, they described a new form of trauma; the trauma of being prevented from informally debriefing with colleagues face‐to‐face in the workplace.

#### 4.4.4 Managing Trauma After the Event: ‘If Something Bad Happens Whilst You're on Shift, You Just Have to Pretend it Hasn't Happened’

Many participants talked about how, during the COVID‐19 pandemic, their trauma became cumulative, especially when they perceived that the event that caused the trauma had never been dealt with at the time that it occurred. This was particularly evident for participants working in areas where trauma is more commonly experienced, such as emergency departments. The following participant describes how there is an expectation that nurses will immediately move on from a traumatic event to continue performing their role:

… as nurses … I don't know if disassociate is the right word, but we're kind of taught we have to do that. If something bad happens whilst you're on shift, you just have to pretend it hasn't happened and keep going, especially in emergency because if something really bad can happen, someone could die … and then 10 min later, especially in these smaller places, there's someone else at the door whose half nearly dead, you know, and you've just got to turn off from the last one and go on to the next one. There's no real healthy space to actually process and deal with it … and by that time, you've really kind of shoved it into a box and like, ‘oh, it's fine, it's over and done with’ … so you just put it in the box and move on. (Participant 26)

Participants identified a form of trauma from these accumulated episodes, especially where formal debriefing was lacking or absent. The informal debriefing among the team previously identified as a critical factor for processing such experiences is not always experienced positively. The expectation that colleagues who shared the trauma will provide support to each other following a traumatic event is described by the following participant as non‐existent and as such, a source of exacerbation of trauma:

I think the word trauma is actually appropriate because … we can be involved in traumatic instances but then there's the what happens afterwards … we often think that we work in healthcare and there's this belief that it's a caring environment and we care for patients we should care for each other, support each other … the experience from each other isn't that you're being supported after facing a traumatic instance and it's like what happens afterwards is actually worse than the trauma that you've been through or the instance that we found traumatic. (Participant 36)

In some cases, nurses needed to change roles to manage the traumatic stress of the COVID‐19 pandemic. Many nurses who described their traumatic stress during the COVID‐19 pandemic had insight into the impact it was having on their health and well‐being and reported making changes to lifestyle, including managing their risky alcohol consumption habits, changing jobs, either moving internally in their organisation or leaving their organisation or profession entirely. Participants reported that many colleagues simply retired early. Some participants described their experiences of trauma in previous roles resulted in instigating a program of support for staff to facilitate semi‐formal debriefing or counselling sessions. As the following participant notes, these are usually part of the services usually offered to patients:

In a different sense, our organisation is pushing trauma‐informed care towards our patients. And I thought I'd utilise that, even towards our own employees, the importance of it and unpacking that. So, I used that as a bit of an in for getting the social workers around. (Participant 43)

Finally, as the following exemplar indicates, the trauma of the COVID‐19 pandemic was described as prevalent among nurses:

… COVID has definitely been a topic that's been brought up by staff. As, yeah, I guess a trigger and a thing that they're aware of in the last 12 months. But that's something that I've kind of initiated as opposed to, as being advised for me to provide to nursing staff. And then I've seen them having those discussions in the handover room, out on the floor. Just being more open about it and talking about it, which is kind of the first step to people dealing with it. (Participant 43)

## 5 Discussion

When the COVID‐19 pandemic was declared in Australia, there was a hiatus before the reality of the consequences impacted our health care system (Berger and Reupert 2020). When it did, it is reasonable to say that the healthcare system was not prepared for what was to ensue. This was evidenced by the lack of basic resources in sufficient volume to meet the needs of the rapidly escalating disaster, compounded by the lack of sound epidemiological information on how to manage and contain the spread of the virus, and treat those who originally contracted COVID‐19 (Nadkarni et al. 2020; Podubinski et al. 2021; Tran et al. 2022). The nurses interviewed for this study described experiencing harms caused directly and indirectly by their experiences of the COVID‐19 pandemic, which were described by these participants as traumatic. These traumas were many and variable and presented a picture of a caring workforce that was overwhelmingly affected and impacted by a phenomenon that has not been encountered before in modern healthcare environments. Our findings are supported by a growing body of work that indicates the mental and physical health impact of working as a nurse during the pandemic (Galanis et al. 2021). Furthermore, our findings indicate that although nurses were portrayed as ‘heroes’ during the pandemic, for many participants there was a high personal cost of working as a nurse throughout COVID‐19 (Mohammed et al. 2021).

Our findings show participants are part of a nursing workforce that is exhausted and burnt‐out, having experienced traumatic stress related to their past experiences in managing in healthcare settings during the COVID‐19 pandemic. Furthermore, these findings indicate a workforce that is potentially at risk of leaving the profession, as found in the work of Raso et al. (2021), whose survey of 5088 registered nurses in the United States found that 30.5% rated the pandemic at 10 for impact (on a scale of 0 to 10). Those who rated the pandemic at this level had a higher intention to leave their roles, indicating that those who describe a high level of impact (including traumatic stress) are more inclined to leave. In our study, traumatic stress and trauma to participants were heightened by the constantly changing clinical and infection control information as COVID‐19 spread throughout the country; the fear associated with the demonstrated virulence and deadly effects of a virus that nurses were forced to face and manage, fears that never left them and were triggered by post‐COVID outbreaks; and finally, the disruption to the bonding between and among nurses that occurs and is rarely or seldom identified as a critical part of coping and managing the often traumatic events that regularly occur in nursing.

Participants reported a myriad of circumstances that caused them substantial traumatic stress during this time, from being without sufficient personal protective equipment (PPE) to daily changes in what PPE was considered safe to manage the virus. Both situations were described as causing fear, and therefore traumatic stress. These findings echo the work of Dempster et al.'s (2024) qualitative study of 21 emergency department nurses in a private hospital setting in Melbourne, Australia, where participants highlight a shortage of PPE, uncertainty on changing equipment settings and significant discomfort in working nursing shifts in protective equipment. Jose et al. (2021) also provide evidence to support the problematic nature of prolonged use of PPE, including headache, sweating and difficulty breathing. For our participants, these factors compound an already stressful work environment.

Participant reports of factors such as fear and the rapidly changing situation causing traumatic stress are consistent with large studies conducted using other methodologies. For example, the work of Pascoe et al. (2022), whose cross‐sectional survey shows that mental health symptoms, including post‐traumatic stress disorder, anxiety and burnout, were significantly more prevalent in a cohort of nurses (3082), when compared to other healthcare workers (4763), especially where nurses had exposure to the COVID‐19 virus. These findings are also echoed by Dobson et al. (2021), who noted that psychological distress such as post‐traumatic stress symptoms was also prevalent in a smaller cohort of 320 healthcare workers in a major tertiary hospital in Melbourne, Australia.

Our study is the first to provide a rich description of the traumatic experiences of nurses in Australia during the pandemic using a qualitative methodology. Our work differs from other survey work, such as Foli's et al. (2021) qualitative survey of 105 nurses in the United States as we have utilised an in‐depth interview to collect rich data on the effects of the pandemic on participants. Nurses in our study describe experiences that are consistent with PTSD, even going so far as to use this term to describe these occurrences in themselves. This is consistent with other studies of healthcare workers where PTSD rates were high and recommendations in mitigating this with psychological interventions were made (Aggar et al. 2022; d'Ettorre et al. 2021).

Traditionally, one of the ways nurses have managed workplace stress is to debrief informally with each other (Zheng et al. 2018), and our study found that the inability to do this was described as extremely detrimental to participant mental health. While there is evidence to support the importance of formalised debriefing of post‐traumatic experiences in nurses (Evans et al. 2023), and further evidence to support the need for debriefing after every critical incident (Scott et al. 2022), there is a dearth of evidence on the benefits of informal debriefing within this population. The COVID‐19 pandemic restrictions on face‐to‐face contact in many parts of Australia resulted in an inability for nurses to use their informal de‐stress techniques; additionally, it also resulted in a significant increase in the number of stressful and traumatic events they were dealing with.

## 6 Strengths and Limitations

To our knowledge, this is the first Australian study using in‐depth qualitative methods to explore nurses' perceptions and experiences of their perceived trauma through, and beyond, the COVID‐19 pandemic. As with all studies, there are limitations that need to be considered when interpreting these findings. The sampling methodology used to recruit nurses to the parent survey relied heavily on distribution through professional associations; arguably, this method may have resulted in an underrepresentation of less experienced, younger nurses who may not yet be engaged with their professional nursing association. Male nurses are slightly underrepresented in the sample at 10%, with the latest Australian nursing registration data indicating a percentage of males in the profession at approximately 13% (Australian Government Department of Health 2024). The project itself is cross‐sectional in nature and represents participant opinions at the time of interview rather than longitudinally. In addition, data is subjective and, like all qualitative studies, not generalisable to the wider nursing workforce. However, our findings present an important insight into the well‐being of nurses in a post‐pandemic world and have important implications for how the mental health of the nursing workforce should be protected and promoted to ensure resilience for future significant events.

## 7 Conclusion

The COVID‐19 pandemic has had a significant impact on nursing workforces globally, and despite a lower mortality rate than that experienced in other countries, Australia is no exception. Nurses have experienced events they describe as traumatic, and although the pandemic was declared over, this trauma persists and has a strong impact on the well‐being of the nursing workforce. It is imperative that nursing organisations globally address the trauma experienced by the nursing workforce to address high rates of turnover intention among nurses, poor workforce well‐being, and ultimately, to ensure a sustainable workforce that provides safe, quality care to all healthcare consumers.

## 8 Relevance to Clinical Practice

The nursing workforce has undergone significant challenges over the past 4 years, related to factors stemming from the COVID‐19 pandemic: continual use of personal protective equipment (PPE), loss of colleagues to illness and death, resourcing challenges, and as this paper indicates, vicarious trauma. As research indicates, poor mental health among caregivers such as nurses' results in suboptimal care as well as challenges to recruitment, retention and workforce sustainability. It is imperative that threats to well‐being and mental health among nurses are explored and addressed to ensure patients and healthcare consumers receive quality and safe nursing care.

## Author Contributions

Each author certifies that their contributions to this work meet the standards of the International Committee of Medical Journal Editors.

## Conflicts of Interest

The authors declare no conflicts of interest.

## Data Availability

The data that support the findings of this study are available on request from the corresponding author. The data are not publicly available due to privacy or ethical restrictions.
